# Severity and Outcome of Post-Vaccine COVID-19 among Healthcare Workers in a University Hospital in India

**DOI:** 10.25122/jml-2023-0017

**Published:** 2023-05

**Authors:** Jagdish Vishnoi, Rajendra Kumar Sharma, Japan Patel, Jagdish Chandra Sharma, Kalu Ram Sharma, Urvansh Mehta

**Affiliations:** 1. Department of Medicine, Pacific Medical College and Hospital, Pacific Medical University, Udaipur, Rajasthan, India; 2. Department of Pediatrics, Pacific Medical College and Hospital, Pacific Medical University, Udaipur, Rajasthan, India

**Keywords:** Healthcare workers, SARS-CoV-2 infection, Covishield, post-vaccine COVID-19, breakthrough cases, booster dose

## Abstract

Healthcare workers (HCWs) are at high risk of COVID-19 infection despite vaccination. Limited data exist on COVID-19 cases among vaccinated HCWs. This study aimed to describe the clinical characteristics and outcomes of RT PCR-confirmed COVID-19 cases in vaccinated HCWs, at a COVID clinic in a medical college hospital. This single-center, prospective cohort study included HCWs who received at least one dose of the COVID-19 vaccine and tested positive for COVID-19 within 6 months. Data on demographics, symptoms, work category, COVID-19 vaccination interval, and infection severity were collected. Of 2381 vaccinated HCWs, 105 tested positive and were categorized as mild, moderate, or severe cases. Among vaccinated HCWs, 4.41% had post-vaccine COVID-19 infections. All 105 cases received the first dose, and 79 received the second dose. Of the cases, 47.6% were partially vaccinated, and 53.3% were breakthrough cases. The mean age was 30.90±8.69 years, with 63.8% male and 36.2% female cases. Most cases (85.7%) acquired infection in the hospital, and 47.6% had direct contact with COVID-19 patients. Common symptoms included fatigue (85.7%), fever (82.9%), and cough (64.8%). Among cases, 93.3% were mild, 5.7% were moderate, and 0.9% were severe. Hospital admission and supplemental oxygen therapy were required for moderate and severe cases. No mortality was reported. Certain variables were associated with age, preventive measures, workplace type, symptoms, and comorbidities. Breakthrough infections can occur among fully vaccinated HCWs but with reduced severity and mortality. Monitoring and infection control measures remain crucial even in vaccinated individuals. This study provides insights into clinical presentations, oxygen therapy requirements, and outcomes of post-vaccine COVID-19 cases among HCWs. The data will inform strategies for booster doses to prevent COVID-19.

## INTRODUCTION

Kerala, India, was the site of the first outbreak of Coronavirus Disease-19 (COVID-19) infection [1] brought on by the severe acute respiratory syndrome coronavirus 2 (SARS-Cov-2). As of November 1, 2022 [2], there have been almost 44.6 million infections and 0.53 million fatalities, with a nationwide mortality rate of 1.2%. Healthcare professionals (HCWs) and healthcare services in India have faced significant strain in dealing with this pandemic. HCWs who come into contact with infected or suspected patients have an increased risk of developing SARS-CoV-2 infection [3].

While fever, exhaustion, dry cough, myalgia, and dyspnea are the most common presenting symptoms, some individuals also reported experiencing diarrhea, nausea, vomiting, and new-onset anosmia or ageusia. Most SARS-CoV-2-infected people (between 75 and 80%) remain asymptomatic but can still transmit the infection [4]. Acute respiratory distress syndrome (ARDS), multi-organ dysfunction syndrome (MODS), mild symptoms, pneumonia with varied severity, and mortality have all been reported as clinical manifestations of COVID-19 [5,6]. The unique transmission capabilities of SARS-CoV-2 and the lack of effective antiviral medications have contributed to the widespread spread and severity of the disease.

The incidence of COVID-19 among HCWs varies considerably around the globe, from 5 to 45 percent. This wide range can be attributed to institutional work-based protocols for SARS-CoV-2 prevention and local guidelines for the use of personal protective equipment. For example, the incidence rate is 5.3 percent at Washington University Hospital and 12 percent in New Jersey [7]. A recent study found that 44 percent of 200 HCWs tested positive for SARS-CoV-2 infection using RT-PCR at some point [8]. HCWs working in wards or intensive care units are at risk of exposure to COVID-19 patients or contaminated objects, which can result in secondary transmission to patients, family members, and the general public. While some individuals may experience mild to moderate symptoms, others may develop severe complications such as ARDS and MODS, leading to mortality.

Evidence suggests that HCWs are particularly vulnerable to SARS-CoV-2 infection due to repeated occupational exposure [9]. Therefore, additional intervention strategies are needed to protect HCWs at a higher risk [10]. The development and distribution of a safe and effective COVID-19 vaccine are crucial steps in containing the epidemic and safeguarding HCWs. Several vaccines, including Pfizer, Moderna, Sputnik, and Oxford-AstraZeneca, have been approved by the World Health Organization (WHO) to combat the pandemic. Currently, numerous vaccinations are being tested in clinical studies worldwide.

In India, the deployment of the Oxford AstraZeneca ChAdOx1 (Covishield) adenovirus vector vaccine and the Bharat Biotech vaccine, Covaxin, began on January 16, 2021. India has achieved rapid rollout of COVID-19 vaccinations, with the Serum Institute of India in Pune manufacturing the Oxford AstraZeneca vaccine locally. Covishield was the initial vaccine administered to HCWs during the early stages of vaccination. The first round of immunization with the novel vaccine provides an opportunity to assess the efficiency of the vaccine in preventing symptomatic COVID-19, reducing disease severity, and evaluating its performance in real-world settings [11]. It is important to note that protection from COVID-19 infection does not occur immediately after immunization and takes time to develop. Three to four weeks after receiving a single dose of the AstraZeneca vaccine, vaccination efficacy (VE) for preventing COVID-19 infection is expected to be between 60 to 80% [12]. After the second dose, VE reaches a peak of above 85%. It is important to note that no vaccination is 100% effective, and it is still possible to contract the virus even after vaccination.

Following immunization, there have been fewer confirmed cases of COVID-19, and the severity of the infection tends to be milder. Asymptomatic or mildly symptomatic cases are anticipated throughout the post-vaccination interval. It is crucial to investigate the clinical characteristics of COVID-19 cases among healthcare workers (HCWs) following immunization, including the risk of infection, potential sources of infection, and exposure information.

Few studies examine the clinical characteristics of HCWs with COVID-19, mostly focusing on personal safety and mental health concerns. Particularly, there are no published Indian data on post-vaccination COVID-19 cases, which include demographics, clinical characteristics, the severity of the disease, and outcomes in healthcare professionals. Therefore, conducting research on confirmed post-vaccine COVID-19 cases among HCWs is necessary to gather knowledge and data on the effectiveness of the vaccine in preventing severe infections.

This study aimed to identify the predictors of severity and outcomes of post-COVID-19 vaccine among HCWs and assess COVID-19 vaccine effectiveness (VE).

## Material and Methods

### Study design and setting

This 6-month prospective cohort study was conducted at a single university hospital in Rajasthan, India, to investigate the severity and outcomes of post COVID-19 vaccine among healthcare workers (HCWs) and assess the effectiveness of the COVID-19 vaccine. The study included HCWs working at a university hospital who received the SARS-CoV-2 vaccine between January 23, 2021, and August 31, 2021. The study analyzed the results of post-vaccination COVID-19 cases confirmed through RT-PCR, focusing on the severity of the disease, comorbidities, and the vaccine's efficacy among HCWs. Following the administration of the second dose of the vaccine, the participants were monitored for six months to assess their post-vaccination outcomes.

Inclusion Criteria

- Healthcare workers (HCWs) who received at least the first dose of the COVID-19 vaccine.
- HCWs who tested positive for COVID-19 after vaccination using RT-PCR.

Exclusion Criteria

- HCWs who tested positive for COVID-19 before receiving the vaccination.
- Unvaccinated HCWs working in COVID-19 areas.
- HCWs who were not working in COVID-19 areas.

The main goal of the study was to determine the exposure and origin of SARS-CoV-2 infection among HCWs who received partial or complete immunization against SARS-CoV-2 within the first six months following immunization. The nasopharyngeal swabs from these HCWs were collected and forwarded to the in-hospital bio-safety level-2 NABL accredited virology lab to perform the SARS-CoV-2 RT PCR test, regardless of whether they had received the first or second dose of the vaccination. A SARS-CoV-2 RT PCR positive was referred to as "post-vaccine COVID-19." Therefore, under the current study procedure, these were classified as "cases".

### Data Collection

Data were collected from the cases in the COVID clinic who had received at least the first dose of the vaccine and developed symptoms within the first six months of the post-vaccination period. The sample size for this study was 105 out of 2879 eligible cases.

Clinical evaluation was performed, including respiratory rate, temperature, blood pressure, O_2_ saturation, and clinical symptoms. Detailed information was obtained on demographics, source of contact, direct or indirect exposures to SARS-CoV2, medical and vaccination history, workplace, clinical illness-symptomatology, outpatient visits or hospitalizations related to the current illness episode, and comorbid conditions. Based on the severity of COVID-19, cases were categorized as mild, moderate, or severe, and these details were recorded in the patient proforma.

### Key definitions

The following definitions were used to describe COVID-19 infection following vaccination:

1. Post-vaccine COVID-19 HCW case: A "HCW case" is defined as a healthcare worker who received either one or two doses of the SARS-CoV-2 vaccination and had at least one positive SARS-CoV-2 RT PCR test result during the research period.

2. Cases that developed symptoms between 0 and 15 days after receiving the first dose of a COVID-19 vaccine were not protected by immunization. These people are not regarded as immune from vaccination since the time since vaccination was not long enough to generate immunity.

3. Partially vaccinated case: Individuals whose first symptoms appeared between 0 and 29 days after receiving the second dose of the COVID-19 vaccine in a two-dose series or within 15 days of the first dose. These people were not considered fully protected since they had not yet received the second dosage or had only recently received the second dose, even though this time interval following vaccination might have been adequate to acquire some immunity.

4. Breakthrough/fully vaccinated (immunized) case: People whose first symptoms appeared 29 days after receiving the COVID-19 vaccine's second dosage. These people are thought to be completely protected against vaccination, but since vaccine efficacy is not 100%, it is expected that some people may still contract the disease after receiving the full course of treatment.

## STATISTICAL ANALYSIS

The primary analysis included all confirmed post-vaccine COVID-19 cases among HCWs in our university hospital. The secondary analyses focused on comparing the clinical profile of individuals who received 2 doses of the vaccine versus those who received only 1 dose, stratified by (1) symptoms and clinical profile and (2) hospitalized and non-hospitalized (home quarantine) cases. For continuous variables, data were presented as mean and standard deviation (SD), and for categorical variables, frequency (number and percentage). Student's t-test for continuous variables and Chi-square test for categorical variables were used to compare COVID-19 patients who required hospital admission vs. home quarantine. The estimation of all quantitative variables, including age, was done using measures of central position (mean). Qualitative or categorical variables were described in terms of proportions. The impact of comorbidities (diabetes mellitus and hypertension) on the severity of COVID-19 was examined using univariate and multivariate logistic regression analysis. Since mortality was 0, it was impossible to utilize it as an outcome metric.

## RESULTS

There were 3263 HCWs in the hospital, of which 2879 (88.23%) were registered for the first phase of the vaccination drive. 82.70% (2381) of the registered HCWs got the first dose of the COVID-19 vaccine. These 2381 vaccinated HCWs were followed for 6 months from the date of the first dose of vaccine for post-vaccine COVID-19 symptoms and subsequently underwent RT-PCR tests for confirmation per the institutional guidelines. We found that 4.41% of the HCWs had COVID-19 infections post-vaccine within 6 months of the first vaccination dose (Table 1).

**Table 1.** HCWs distribution in terms of COVID-19 status post-vaccine (n=2381)

Vaccinated HCWs	
1^st^ Dose 2^nd^ Dose	2381 (100%)
Post-COVID vaccine cases	105 (4.41%) CI ▢3.6 – 5.3%
Case Distribution (n=105)	
Mild Moderate Severe Death	98 (93.33%) 6 (5.71%) 1 (0.95%) 0 (0.00%)

**Figure 1.**
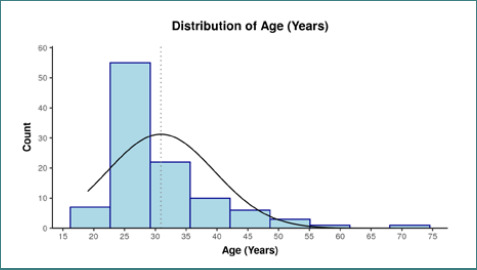
Distribution of age among cases

**Figure 2.**
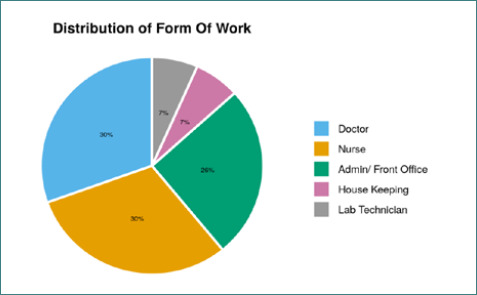
Distribution of work category among cases

**Figure 3.**
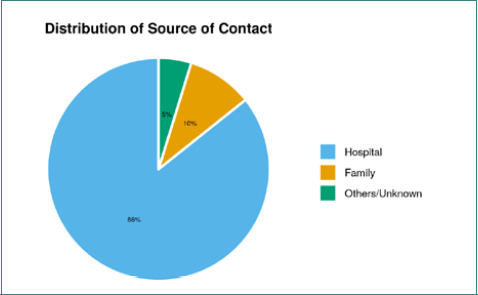
Association between contact/source and infection

The mean age of participants was 30.90 ± 8.69 years, and the median age was 29.00 years (IQR= 25.00-33.00). The variable age in years was not normally distributed (Shapiro-Wilk Test: p=<0.001) (Figure 1). Age ranged from 19 – 69 years. Sixty-seven (63.8%) cases were male, while 38 (36.2%) were female. Doctors, nurses, housekeeping workers, and lab technicians were considered on the frontline of the HCWs group. 78 (74.3%) cases were from the frontline HCWs group, while the non-frontline HCWs group had 27 (25.7%) cases (Figure 2). The majority (90; 85.7%) of the cases acquired infection from the hospital (Table 2, Figure 3).

**Table 2.** Column I. Demographic and clinical characteristics of the cases

Characteristics	Frequency (%)	95% CI
**Age (years) mean ± SD**	30.90±8.69	
**Gender** Male Female	67 (63.8%) 38 (36.2%)	
**Form of work**		
Doctor	32 (30.5%)	
Nurse	32 (30.5%)	
Admin/ Front Office	27 (25.7%)	
House Keeping	7 (6.7%)	
Lab Technician	7 (6.7%)	
**Source of contact**		
Hospital	90 (85.7%)	
Family	10 (9.5%)	
Others/Unknown	5 (4.8%)	
**Preventive measures: N-95 Mask (Yes)**	91 (86.7%)	
**Preventive measures: Surgical mask (Yes)**	24 (22.9%)	
**Preventive measures: Other (Yes)**	6 (5.7%)	
**Type of workplace**		
COVID Area (Clinical)	45 (42.9%)	
Non-COVID (Non-clinical)	39 (37.1%)	
Non-COVID (Clinical)	16 (15.2%)	
COVID Area (Non-clinical)	5 (4.8%)	
**Vaccine: 1^st^ dose (taken)**	105 (100.0%)	95.6%-100.0%
**Vaccine: 2^nd^ dose**		
Taken	79 (75.2%)	65.7%-82.9%
Not Taken	26 (24.8%)	17.1%-34.3%
**Vaccine-infection interval**		
0-14 Days	20 (19.0%)	
15-28 Days	6 (5.7%)	
29-56 Days	23 (21.9%)	
>56 Days	56 (53.3%)	
**Symptoms: Fever (Yes)**	87 (82.9%)	
**Symptoms: Cough (Yes)**	68 (64.8%)	
**Symptoms: Breathlessness (Yes)**	13 (12.4%)	

**Table 2.** Column II. Demographic and clinical characteristics of the cases

Characteristics	Frequency (%)	95% CI
**Symptoms: Fatigue (Yes)**	90 (85.7%)	
**Symptoms: Anosmia (Yes)**	20 (19.0%)	
**Symptoms: Diarrhoea (Yes)**	20 (19.0%)	
**Symptoms: Throat Pain (Yes)**	4 (3.8%)	
**Symptoms: No Symptoms (Yes)**	1 (1.0%)	
**Place of management**		
Home Quarantine	98 (93.3%)	
Hospital	7 (6.7%)	
**Antivirals (Yes)**	68 (64.8%)	
**Comorbidities**		
None	97 (92.4%)	85.1%-96.4%
Hypothyroidism	3 (2.9%)	0.7%-8.7%
Diabetes	2 (1.9%)	0.3%-7.4%
HTN	2 (1.9%)	0.3%-7.4%
Asthma	1 (1.0%)	0.0%-6.0%

All the cases used face masks as a protective measure to prevent infection. Specifically, 91 cases (86.7%) used N95 masks, while 24 (22.9%) used surgical masks. Some patients used both N95 and surgical masks. Less than half (50; 47.6%) of the cases had direct contact with COVID-19 patients or their secretions/nasopharyngeal samples while rendering their services in COVID areas, despite using PPE kits and taking all necessary precautions to prevent infection. On the other hand, 55 cases (52.4%) were not directly involved in patient care services.

**Figure 4.**
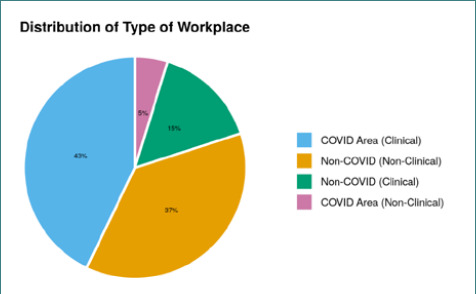
Association between the workplace and infection

All 105 cases (100.0%) had received their first dose of the COVID-19 Covishield vaccine. Among them, 79 cases (75.2%) had also received the second dose of the same vaccine, while 26 cases (24.8%) had not yet received the second dose. This indicates that the majority of cases received both doses of the vaccine (75.2%), while a smaller proportion received only the first dose (24.8%). When analyzing the interval between vaccination and infection, 20 cases (19.0%) had an interval of 0-14 days, 6 cases (5.7%) had an interval of 15-28 days, 23 cases (21.9%) had an interval of 29-56 days, and the majority, 56 cases (53.3%), had an interval of more than 56 days. This information is presented in Table 3, Figure 4.

**Table 3.** Association between the form of work and clinical-demographic characteristics

Characteristics	Form of work					
Doctor (n = 32)	Nurse (n = 32)	Admin/ Front Office (n = 27)	House Keeping (n = 7)	Lab Technician (n = 7)	p-value
Age (Years)***	31.31±11.02	29.50±6.03	29.41±5.97	42.14±6.36	30.00±11.34	0.014^1^
**Gender**						0.081^2^
Male	21 (65.6%)	25 (78.1%)	16 (59.3%)	2 (28.6%)	3 (42.9%)	
Female	11 (34.4%)	7 (21.9%)	11 (40.7%)	5 (71.4%)	4 (57.1%)	
**Source of contact**						0.123^2^
Hospital	28 (87.5%)	30 (93.8%)	19 (70.4%)	6 (85.7%)	7 (100.0%)	
Family	2 (6.2%)	1 (3.1%)	7 (25.9%)	0 (0.0%)	0 (0.0%)	
Others/Unknown	2 (6.2%)	1 (3.1%)	1 (3.7%)	1 (14.3%)	0 (0.0%)	
Preventive measures: N-95 Mask (Yes)***	31 (96.9%)	30 (93.8%)	19 (70.4%)	6 (85.7%)	5 (71.4%)	0.012^2^
**Preventive measures: Surgical Mask (Yes)**	7 (21.9%)	4 (12.5%)	11 (40.7%)	1 (14.3%)	1 (14.3%)	0.114^3^
Preventive measures: Other (Yes)***	1 (3.1%)	0 (0.0%)	5 (18.5%)	0 (0.0%)	0 (0.0%)	0.044^2^
Type of workplace***						<0.001^3^
COVID Area (Clinical)	17 (53.1%)	25 (78.1%)	0 (0.0%)	1 (14.3%)	2 (28.6%)	
Non-COVID (Non-Clinical)	7 (21.9%)	1 (3.1%)	24 (88.9%)	3 (42.9%)	4 (57.1%)	
Non-COVID (Clinical)	8 (25.0%)	6 (18.8%)	0 (0.0%)	1 (14.3%)	1 (14.3%)	
COVID Area (Non-Clinical)	0 (0.0%)	0 (0.0%)	3 (11.1%)	2 (28.6%)	0 (0.0%)	
**Vaccine: 1^st^ dose (taken)**	32 (100.0%)	32 (100.0%)	27 (100.0%)	7 (100.0%)	7 (100.0%)	1.000^3^
**Vaccine: 2^nd^ dose**						0.400^3^
Taken	26 (81.2%)	22 (68.8%)	19 (70.4%)	5 (71.4%)	7 (100.0%)	
Not Taken	6 (18.8%)	10 (31.2%)	8 (29.6%)	2 (28.6%)	0 (0.0%)	
**Vaccine-infection interval**						0.115^3^
0-14 Days	5 (15.6%)	6 (18.8%)	8 (29.6%)	1 (14.3%)	0 (0.0%)	
15-28 Days	2 (6.2%)	0 (0.0%)	3 (11.1%)	0 (0.0%)	1 (14.3%)	
29-56 Days	11 (34.4%)	3 (9.4%)	5 (18.5%)	1 (14.3%)	3 (42.9%)	
>56 Days	14 (43.8%)	23 (71.9%)	11 (40.7%)	5 (71.4%)	3 (42.9%)	
**Symptoms: Fever (Yes)**	25 (78.1%)	30 (93.8%)	21 (77.8%)	5 (71.4%)	6 (85.7%)	0.251^2^
**Symptoms: Cough (Yes)**	20 (62.5%)	23 (71.9%)	18 (66.7%)	4 (57.1%)	3 (42.9%)	0.634^2^
**Symptoms: Breathlessness (Yes)**	4 (12.5%)	5 (15.6%)	2 (7.4%)	1 (14.3%)	1 (14.3%)	0.861^2^
**Symptoms: Fatigue (Yes)**	26 (81.2%)	28 (87.5%)	22 (81.5%)	7 (100.0%)	7 (100.0%)	0.718^2^
**Symptoms: Anosmia (Yes)**	8 (25.0%)	6 (18.8%)	5 (18.5%)	0 (0.0%)	1 (14.3%)	0.646^3^
**Symptoms: Diarrhoea (Yes)**	9 (28.1%)	7 (21.9%)	3 (11.1%)	0 (0.0%)	1 (14.3%)	0.316^3^
Symptoms: Throat Pain (Yes)***	0 (0.0%)	0 (0.0%)	2 (7.4%)	0 (0.0%)	2 (28.6%)	0.010^2^
**Symptoms: No Symptoms (Yes)**	1 (3.1%)	0 (0.0%)	0 (0.0%)	0 (0.0%)	0 (0.0%)	1.000^2^
**Place of Management**						0.200^2^
Home Quarantine	29 (90.6%)	31 (96.9%)	26 (96.3%)	7 (100.0%)	5 (71.4%)	
Hospital	3 (9.4%)	1 (3.1%)	1 (3.7%)	0 (0.0%)	2 (28.6%)	
**Anti-Viral (Yes)**	21 (65.6%)	25 (78.1%)	14 (51.9%)	3 (42.9%)	5 (71.4%)	0.185^2^
Comorbidities***						0.002^2^
None	28 (87.5%)	32 (100.0%)	27 (100.0%)	4 (57.1%)	6 (85.7%)	
Hypothyroidism	0 (0.0%)	0 (0.0%)	0 (0.0%)	2 (28.6%)	1 (14.3%)	
Diabetes	1 (3.1%)	0 (0.0%)	0 (0.0%)	1 (14.3%)	0 (0.0%)	
HTN	2 (6.2%)	0 (0.0%)	0 (0.0%)	0 (0.0%)	0 (0.0%)	
Asthma	1 (3.1%)	0 (0.0%)	0 (0.0%)	0 (0.0%)	0 (0.0%)	

***Significant at p<0.05, ^1^Kruskal Wallis Test, ^2^Fisher's Exact Test, ^3^Chi-Squared Test

**Figure 5.**
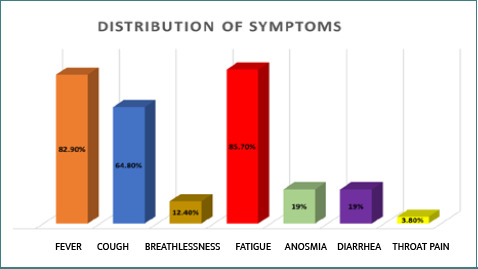
Frequency of symptoms with infection

The most common symptom among cases was fatigue (85.7%), followed by fever (82.9%) and cough (64.8%) (Figure 5). Most cases experienced one or more symptoms, with only one being asymptomatic. Out of the total cases, 68 (64.8%) were prescribed antiviral medications, while 37 (35.2%) did not receive antiviral treatment. Only 8 (7.6%) cases had comorbidities, including hypothyroidism 3 (2.9%), diabetes mellitus (1.9%), hypertension 2 (1.9%), and asthma 1 (1.0%). Hypothyroidism was the most common comorbidity, followed by diabetes and hypertension (Table 3).

According to the case-category definition of COVID-19 severity, a significantly higher number of cases (98; 93.3%) were classified as mild cases and were managed through home quarantine. Only a small number of cases (7; 6.7%) required hospital admission. Among the hospitalized cases, 6 were classified as moderate, and 1 was classified as severe. All hospitalized patients required supplemental oxygen therapy. No deaths were reported among the home-quarantined and hospitalized cases (Table 1).

Significant associations (p<0.05) were observed between the form of work and several factors, including age (years), preventive measures (N-95 mask and other), workplace type, throat discomfort, and comorbidities (Table 3).

The chi-square test was used to investigate the relationship between the form of work and the type of workplace. The distribution of type of workplace varied significantly among the different groups (χ^2^=75.953, p<0.001). The strength of association between the two variables, as measured by Cramer's V, was found to be 0.49, indicating a moderate association. The bias-corrected Cramer's V also revealed a moderate strength of association between the variables, with a value of 0.46 (Tables 4.1, 4.2, and 4.3).

**Table 4.1.** Association between the form of work and type of workplace (n = 105)

Characteristics	Form of work						Chi-Square Test	
Doctor	Nurse	Admin/ Front Office	House Keeping	Lab Technician	Total	X2	p value
COVID Area (Clinical)	17 (53.1%)	25 (78.1%)	0 (0.0%)	1 (14.3%)	2 (28.6%)	45 (42.9%)	75.953	<0.001
Non-COVID (Non-Clinical)	7 (21.9%)	1 (3.1%)	24 (88.9%)	3 (42.9%)	4 (57.1%)	39 (37.1%)		
Non-COVID (Clinical)	8 (25.0%)	6 (18.8%)	0 (0.0%)	1 (14.3%)	1 (14.3%)	16 (15.2%)		
COVID Area (Non-Clinical)	0 (0.0%)	0 (0.0%)	3 (11.1%)	2 (28.6%)	0 (0.0%)	5 (4.8%)		
Total	32 (100.0%)	32 (100.0%)	27 (100.0%)	7 (100.0%)	7 (100.0%)	105 (100.0%)		

**Table 4.2.** Association between the form of work and type of workplace (n = 105)

Type of Workplace	Adjusted P Values
COVID Area (Clinical) vs. Non-COVID (Non-Clinical)	<0.001
COVID Area (Clinical) vs. Non-COVID (Clinical)	0.448
COVID Area (Clinical) vs. COVID Area (Non-Clinical)	<0.001
Non-COVID (Non-Clinical) vs. Non-COVID (Clinical)	<0.001
Non-COVID (Non-Clinical) vs. COVID Area (Non-Clinical)	0.337
Non-COVID (Clinical) vs. COVID Area (Non-Clinical)	<0.001

**Table 4.3.** Association between the form of work and type of workplace (n = 105)

Form Of Work	Adjusted P Values
Doctor vs. Nurse	0.052
Doctor vs. Admin/ Front Office	<0.001
Doctor vs. House Keeping	0.028
Doctor vs. Lab Technician	0.248
Nurse vs. Admin/ Front Office	<0.001
Nurse vs. House Keeping	<0.001
Nurse vs. Lab Technician	0.007
Admin/ Front Office vs. Housekeeping	0.029
Admin/ Front Office vs. Lab Technician	0.029
House Keeping vs. Lab Technician	0.755

The odds ratios (OR) for each variable are presented in the "OR (univariable)" column, representing the odds of breakthrough infections when each variable is considered individually (Table 6, Figure 7). The "OR (multivariable)" column displays the odds ratios when all variables are included in the model, controlling for each other. The reference category for each categorical variable is the initial category, and odds ratios are calculated for the other categories. Notably, although not statistically significant, a higher number of breakthrough infections was observed among front-office healthcare workers who directly worked with COVID-19 patients, which may be attributed to the smaller sample size in this subgroup.

**Table 5.** Column I. Association between fatigue and various HCWs characteristics

Characteristics	Symptoms: Fatigue		p-value
Yes (n = 90)	No (n = 15)
**Age (Years)**	30.83±8.96	31.33±7.12	0.535^1^
**Gender**			0.407^2^
Male	56 (62.2%)	11 (73.3%)	
Female	34 (37.8%)	4 (26.7%)	
Form of work			0.718^3^
Doctor	26 (28.9%)	6 (40.0%)	
Nurse	28 (31.1%)	4 (26.7%)	
Admin/ Front Office	22 (24.4%)	5 (33.3%)	
House Keeping	7 (7.8%)	0 (0.0%)	
Lab Technician	7 (7.8%)	0 (0.0%)	

**Table 5.** Column II. Association between fatigue and various HCWs characteristics

Characteristics	Symptoms: Fatigue		p-value
Yes (n = 90)	No (n = 15)
Source of contact***			0.006^3^
Hospital	81 (90.0%)	9 (60.0%)	
Family	5 (5.6%)	5 (33.3%)	
Others/Unknown	4 (4.4%)	1 (6.7%)	
**Preventive measures: N-95 Mask (Yes)**	78 (86.7%)	13 (86.7%)	1.000^3^
**Preventive measures: Surgical Mask (Yes)**	22 (24.4%)	2 (13.3%)	0.511^3^
**Preventive measures: Other (Yes)**	6 (6.7%)	0 (0.0%)	0.590^3^
**Type of workplace**			0.455^3^
COVID Area (Clinical)	40 (44.4%)	5 (33.3%)	
Non-COVID (Non-Clinical)	34 (37.8%)	5 (33.3%)	
Non-COVID (Clinical)	12 (13.3%)	4 (26.7%)	
COVID Area (Non-Clinical)	4 (4.4%)	1 (6.7%)	
**Vaccine: 1^st^ dose (taken)**	90 (100.0%)	15 (100.0%)	1.000^2^
**Vaccine: 2^nd^ dose**			0.194^3^
Taken	70 (77.8%)	9 (60.0%)	
Not Taken	20 (22.2%)	6 (40.0%)	
**Vaccine-infection interval**			0.791^3^
0-14 Days	16 (17.8%)	4 (26.7%)	
15-28 Days	6 (6.7%)	0 (0.0%)	
29-56 Days	20 (22.2%)	3 (20.0%)	
>56 Days	48 (53.3%)	8 (53.3%)	
**Symptoms: Fever (Yes)**	76 (84.4%)	11 (73.3%)	0.284^3^
**Symptoms: Cough (Yes)**	61 (67.8%)	7 (46.7%)	0.113^2^
**Symptoms: Breathlessness (Yes)**	12 (13.3%)	1 (6.7%)	0.687^3^
**Symptoms: Anosmia (Yes)**	18 (20.0%)	2 (13.3%)	0.731^3^
**Symptoms: Diarrhea (Yes)**	20 (22.2%)	0 (0.0%)	0.069^3^
**Symptoms: Throat Pain (Yes)**	4 (4.4%)	0 (0.0%)	1.000^3^
**Symptoms: No Symptoms (Yes)**	0 (0.0%)	1 (6.7%)	0.143^3^
**Place of management**			0.590^3^
Home Quarantine	83 (92.2%)	15 (100.0%)	
Hospital	7 (7.8%)	0 (0.0%)	

**Table 5.** Column III. Association between fatigue and various HCWs characteristics

Characteristics	Symptoms: Fatigue		p-value
Yes (n = 90)	No (n = 15)
**Anti-Viral (Yes)**	61 (67.8%)	7 (46.7%)	0.113^2^
**Comorbidities**			1.000^3^
None	82 (91.1%)	15 (100.0%)	
Hypothyroidism	3 (3.3%)	0 (0.0%)	
Diabetes	2 (2.2%)	0 (0.0%)	
HTN	2 (2.2%)	0 (0.0%)	
Asthma	1 (1.1%)	0 (0.0%)	

***Significant at p<0.05, ^1^Kruskal Wallis Test, ^2^Fisher's Exact Test, ^3^Chi-Squared Test

**Table 6.** Regression analysis of breakthrough infections

Dependent: Infection after 4 Weeks		No	Yes	OR (univariable)	OR (multivariable)
Age (Years)	Mean (SD)	29.3 (7.8)	31.4 (8.9)	1.03 (0.98-1.11, p=0.273)	1.03 (0.96-1.11, p=0.443)
Form Of Work	Doctor	7 (21.9)	25 (78.1)	-	-
	Nurse	6 (18.8)	26 (81.2)	1.21 (0.36-4.25, p=0.756)	1.06 (0.28-4.01, p=0.930)
	Admin/ Front Office	11 (40.7)	16 (59.3)	0.41 (0.13-1.25, p=0.121)	0.52 (0.07-3.26, p=0.496)
	House Keeping	1 (14.3)	6 (85.7)	1.68 (0.23-34.49, p=0.655)	3.44 (0.11-614.48, p=0.554)
	Lab Technician	1 (14.3)	6 (85.7)	1.68 (0.23-34.49, p=0.655)	2.40 (0.24-65.21, p=0.510)
Type of Workplace	COVID Area (Clinical)	8 (17.8)	37 (82.2)	-	-
	Non-COVID (Non-Clinical)	14 (35.9)	25 (64.1)	0.39 (0.14-1.04, p=0.064)	0.51 (0.08-3.31, p=0.457)
	Non-COVID (Clinical)	3 (18.8)	13 (81.2)	0.94 (0.23-4.76, p=0.931)	0.99 (0.22-5.63, p=0.986)
	COVID Area (Non-Clinical)	1 (20.0)	4 (80.0)	0.86 (0.11-18.12, p=0.902)	0.96 (0.05-33.08, p=0.979)
Preventive Measures: N-95 Mask	No	3 (21.4)	11 (78.6)	-	-
	Yes	23 (25.3)	68 (74.7)	0.81 (0.17-2.86, p=0.757)	0.44 (0.08-1.89, p=0.305)
Comorbidities	None	24 (24.7)	73 (75.3)	-	-
	Hypothyroidism	1 (33.3)	2 (66.7)	0.66 (0.06-14.52, p=0.737)	0.17 (0.00-9.27, p=0.372)
	Diabetes	0 (0.0)	2 (100.0)	5145652.86 (0.00-NA, p=0.993)	1793580.98 (0.00-NA, p=0.993)
	HTN	1 (50.0)	1 (50.0)	0.33 (0.01-8.53, p=0.438)	0.32 (0.01-9.73, p=0.461)
	Asthma	0 (0.0)	1 (100.0)	5145652.86 (0.00-NA, p=0.995)	3992385.81 (0.00-NA, p=0.995)

MODEL FIT: X^2^(13) = 10.56, p = 0.648 Pseudo-R^2^ = 0.09 Number in dataframe = 105, Number in model = 105, Missing = 0 AIC = 135, C-statistic = 0.694, H&L = Chi-sq(8) 8.59 (p=0.378)

## DISCUSSION

COVID-19 as a disease and a pandemic has constantly been evolving, demanding an everlasting variable decision-making process via mass education and following a prescribed set of standard operating procedures. Among all, healthcare workers (HCWs) have been at the forefront, facing the highest risk of exposure. Vaccination has played a vital role in curbing the spread of the disease and protecting HCWs from its severe effects. Most individuals diagnosed with COVID-19 have moderate symptoms like fever, coughing, dyspnea, myalgia, and exhaustion. On the other hand, patients with severe instances develop ARDS and serious cardiac and renal consequences, which may result in death [13,14]. A worse prognosis is also linked to advanced age, male gender, and pre-existing chronic illnesses like diabetes, cardiovascular disease, and hypertension [15,16]. There is evidence that vaccines lower symptomatic infection, the severity of sickness, disease mortality, and transmission when COVID-19 cases occur after immunization (post-vaccine COVID-19) [17-19].

Although national statistics on the incidence and mortality of healthcare workers (HCWs) from COVID-19 or post-COVID-19 vaccination in India are lacking, studies from specific regions provide some insights. According to a study in Mumbai by Mahajan *et al.*, HCWs had a prevalence of SARS-CoV-2 infection of 11%, a co-infection rate of 4%, and a fatality rate of 1%. Of the HCWs with COVID-19, 19% reported having comorbidities, with hypertension and diabetes mellitus being the most frequently reported conditions [20]. In a different Indian study, 23.93% of subjects had COVID-19-positive results from PCR testing. This study was conducted to identify the prevalence of COVID-19 among the HCWs working at a gastroenterology department. The results revealed that 23.93% of the subjects tested positive for COVID-19 [21].

The primary objective of this study was to investigate the clinical characteristics and outcomes of healthcare workers (HCWs) who contracted COVID-19 following vaccination. Healthcare providers carry an additional risk of transmitting COVID-19 infection to their patients and the community. Therefore, it is essential to identify the possible sources of infection and understand the clinical presentation in such cases.

Our demographic data is constant with parallel research, including the general population [22]. The most common symptoms observed in our study were fever, cough, and fatigue, which have been consistently observed throughout the pandemic. These symptoms pose a challenge in detecting the disease in its early stages, as they can be mistaken for other viral fevers, leading to delayed diagnosis and potential spread of the virus. Anosmia, previously discussed as a specific feature of COVID-19, was seen in only about 20% of the patients, decreasing its statistical significance [23].

Around 60% of the population in the study comprised doctors and nurses. This high representation of healthcare workers in the study may be attributed to their direct involvement in patient care and increased exposure to the virus. Healthcare workers not only face the physical impact of the disease but also experience a significant mental health burden, which needs to be addressed alongside COVID-19 treatment [24,25]. Firstly, adherence to proper standard operating procedures (SOPs) and infection control practices by healthcare workers may have contributed to reducing the severity of the disease. Additionally, the fact that all patients received at least a single dose of the vaccine may have provided some level of protection against severe illness. Moreover, the relatively younger age group of the patients and a lower prevalence of comorbidities in our study population could have contributed to the milder disease presentation [26].

Although vaccines have shown efficacy in reducing hospitalizations and mortality due to COVID-19, it is important to note that they may not completely prevent infection. Our study revealed that some patients who had achieved full immunization still contracted the infection, resulting in breakthrough cases. This finding is consistent with other studies that have reported a decline in vaccine effectiveness over time [27]. In our study, approximately 53% of patients experienced breakthrough infections after 56 days of receiving the second vaccine dose. This suggests that the protection provided by the vaccines may wane over time. A meta-regression analysis on vaccine effectiveness also supports this observation, indicating that vaccine efficacy against severe COVID-19 declines by approximately 10% after 6 months of complete immunization [28]. Immunological concepts help us understand this phenomenon. Over time, the levels of neutralizing antibodies, which play a crucial role in neutralizing the virus, may decrease effectiveness. This decline in efficacy could be attributed to various factors, including the nature of vaccine-induced antibodies [29,30].

Direct exposure to COVID-19 patients actually increases the risk of infection, as observed in our study. However, it is noteworthy that despite breakthrough infections, most cases experienced mild symptoms or were asymptomatic. This suggests that vaccination plays a crucial role in preventing disease progression to severe illness.

In our study, there was one exceptional case in the severe category, which can be attributed to the patient's age (59 years) and multiple comorbidities. Comorbidities such as uncontrolled diabetes, hypertension, and obesity are known risk factors for severe COVID-19. Our study has shown that half of the population had breakthrough infections. Among the doctors who received two doses of the vaccine, only three required hospitalization, and all had comorbidities.

Administration of booster doses may effectively prevent breakthrough infections for a longer duration and might provide lifelong immunity [31]. Booster dosing has been gaining favorable results in increasing vaccine effectiveness after a complete immunization [32,33]. Studies are required to follow up HCWs after the booster dose to gain further scientific pieces of evidence. Published data show that there is no difference in infection of COVID-19 post-full vaccination and the vaccination scheme [34].

**Figure 6.**
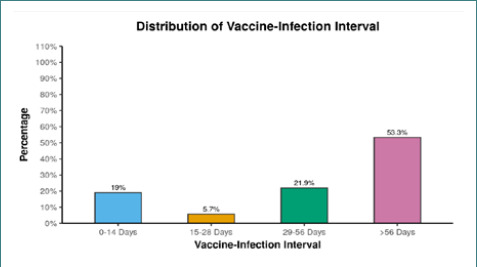
Association of cases with post-vaccine duration

The highest number of infections occurred in the group with a vaccine-infection interval of more than 56 days, accounting for 53.3% of cases. Additionally, 21.9% of participants became infected 29-56 days after receiving the vaccine. These observations suggest a potential decrease in vaccine efficacy over time as the interval between vaccination and infection increases. It is important to note that the infections observed within 0-14 days post-vaccine were relatively higher (19%) compared to the 15-28 days group (5.7%), which may be attributed to the time required for the immune response to develop following vaccination (Figure 6). As there were multiple symptoms, we focused on examining the relationship between fatigue and various other symptoms to assess their significance (Table 5).

**Figure 7.**
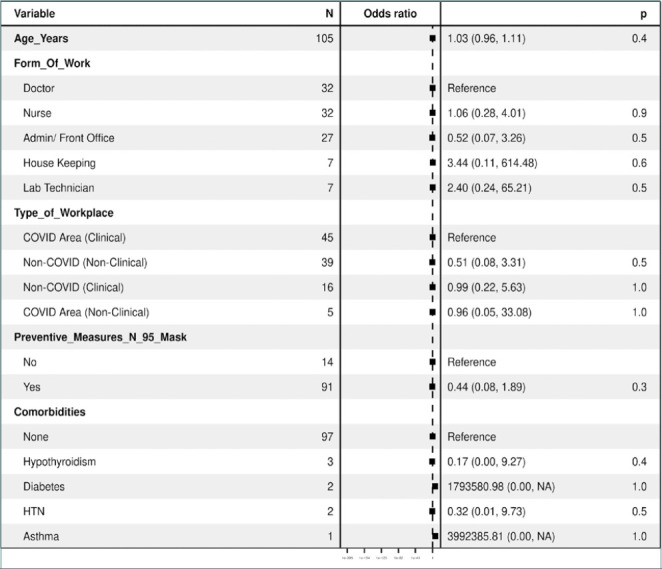
Odds Ratio of Breakthrough Infections

Another study in Israel specifically focused on evaluating breakthrough infections among healthcare workers. The researchers conducted a detailed analysis of healthcare workers who were symptomatic or had known exposure to infection. Of the 12,497 fully vaccinated healthcare workers included in the study, 39 breakthrough infections were observed. Importantly, the majority of these cases were mild to asymptomatic [35]. These findings are consistent with similar studies conducted in Italy and across various European centers. For example, in an Italian study involving 5,996 workers, 582 breakthrough cases were identified, but there were no instances of hospitalization or mortality [36]. Similarly, a study involving 12 different European centers reported 797 breakthrough cases [37].

The presence of a healthy worker effect among healthcare workers (HCWs), a phenomenon in which the index population may not be adequate for comparison with the overall population, posed a challenge in comparing HCWs with the general population.,

The current study is a prospective, 6-month study that included a significantly large cohort of healthcare professionals. To the best of our knowledge, this is the first Indian study among healthcare workers (HCWs) with post-vaccination COVID-19 at a university hospital in Rajasthan, India. The study examined information on clinical presentation, comorbidity, reinfection, treatment response, disease severity, and outcomes. By comparing post-vaccination COVID-19 cases with cases among HCWs who have not received the vaccine, the study is expected to generate valuable data. The interim analysis will provide insights into the effectiveness of the vaccine in reducing disease severity and mortality rates in India.

The current investigation sheds light on the factors that influence the clinical presentation, the need for oxygen therapy, hospital admission, and the outcomes of post-vaccination COVID-19 in HCWs in a sizable cohort. The existing literature offers scant details on comparisons among HCWs with post-vaccination COVID-19 status. In India, there is no direct comparison of the symptomatology and severity of the disease between HCWs and the general population. Our data could be useful in developing strategies to manage the epidemic among HCWs, particularly in how to best use resources for treatment during home isolation as opposed to hospital-based management and the booster dosage plan.

Our study has several limitations that should be considered. Firstly, it was conducted at a single center, which may limit the generalizability of the findings to other healthcare settings. Secondly, we did not perform genome sequencing to confirm the specific SARS-CoV-2 strain involved in the breakthrough infections. We did not measure neutralizing antibody titers, which could provide a more accurate assessment of vaccine efficacy over time. Furthermore, our study did not include an extensive comparative analysis of various symptoms and demographic data. This could have provided a more comprehensive understanding of the clinical presentation and characteristics of breakthrough infections. Lastly, we did not compare the outcomes between vaccinated and unvaccinated individuals, which could have provided valuable insights into the relative effectiveness of the vaccine in preventing infection and disease progression.

## CONCLUSION

After the COVID-19 immunization campaign kicked off on January 23, 2021, and up to August 31, 2021, 2381 HCWs in our hospital received at least one dose of vaccine (Table 1). Out of these, 4.46% (n=105) HCWs got infected (labeled as ‘cases’) within 6 months post-vaccination. 19% (n=20) of cases identified were not yet protected as they got infected within 0 to <15 days after dose 1 of the vaccine administration schedule (Table 2). 27.6%(n=49) became infected when they were partially vaccinated. 53.3% (n=56) became infected when they were fully vaccinated, and these were considered breakthrough cases (Table 2). Based on the COVID-19 case-category definition, 93.3% (n=98), 5.7% (n=6), and 0.9% (n=1) were in the mild, moderate, and severe categories, respectively. All patients from the moderate and severe category required hospital admission and supplemental oxygen therapy. There was zero mortality (Table 1). With the increasing administration of COVID-19 vaccines and the rising incidence of COVID-19 cases, there is a corresponding increase in trends related to partially vaccinated individuals and breakthrough cases. Our findings demonstrate that breakthrough infections can still occur with the 2-dose schedule of Covishield. However, it is noteworthy that the vaccine significantly reduces the severity of the disease, hospitalization rates, and mortality. The results of our study strongly support the role of the COVID-19 vaccine in mitigating moderate to severe illness among vaccinated healthcare workers (HCWs). Based on these findings, we advise individuals who have been vaccinated to continue practicing preventive measures such as regular handwashing, maintaining physical distancing, and wearing masks in public settings. Additionally, we recommend administering booster doses of the vaccine to all HCWs, and the same approach can be extended to the general population.
